# Chromium–Insulin Reduces Insulin Clearance and Enhances Insulin Signaling by Suppressing Hepatic Insulin-Degrading Enzyme and Proteasome Protein Expression in KKAy Mice

**DOI:** 10.3389/fendo.2014.00099

**Published:** 2014-07-07

**Authors:** Zhong Q. Wang, Yongmei Yu, Xian H. Zhang, James Komorowski

**Affiliations:** ^1^Pennington Biomedical Research Center, Louisiana State University System, Baton Rouge, LA, USA; ^2^JDS Therapeutics, LLC, Purchase, NY, USA

**Keywords:** chromium–insulin, IDE, insulin signaling, IRS-1, proteasome, liver

## Abstract

JDS–chromium–insulin (CRI)-003 is a novel form of insulin that has been directly conjugated with chromium (Cr) instead of zinc. Our hypothesis was that CRI enhances insulin’s effects by altering insulin-degrading enzyme (IDE) and proteasome enzymes. To test this hypothesis, we measured hepatic IDE content and proteasome parameters in a diabetic animal model. Male KKAy mice were randomly divided into three groups (*n* = 8/group); Sham (saline), human regular insulin (Reg-In), and chromium conjugated human insulin (CRI), respectively. Interventions were initiated at doses of 2 U insulin/kg body weight daily for 8-weeks. Plasma glucose and insulin were measured. Hepatic IDE, proteasome, and insulin signaling proteins were determined by western blotting. Insulin tolerance tests at week 7 showed that both insulin treatments significantly reduced glucose concentrations and increased insulin levels compared with the Sham group, CRI significantly reduced glucose at 4 and 6 h relative to Reg-In (*P* < 0.05), suggesting the effects of CRI on reducing glucose last longer than Reg-In. CRI treatment significantly increased hepatic IRS-1 and Akt1 and reduced IDE, 20S as well as 19S protein abundance (*P* < 0.01, *P* < 0.05, and *P* < 0.001, respectively), but Reg-In only significantly increased Akt1 (*P* < 0.05). Similar results were also observed in Reg-In- and CRI-treated HepG2 cells. This study, for the first time, demonstrates that CRI reduces plasma insulin clearance by inhibition of hepatic IDE protein expression and enhances insulin signaling as well as prevents degradation of IRS-1 and IRS-2 by suppressing ubiquitin-proteasome pathway in diabetic mice.

## Introduction

Chromium is an essential mineral that appears to have beneficial effects in the regulation of insulin action and in the improvement of carbohydrate and lipid metabolism (1, 2). Studies show that people with type 2 diabetes have lower blood levels of chromium than those without the disease (3). Insulin resistance is the common denominator in a cluster of cardiovascular disease risk factors (4). Chromium picolinate (CrPic) has been shown to reduce insulin resistance and to help reduce the risk of cardiovascular disease and type 2 diabetes (5). It was observed in obese KK/HlJ diabetic mouse model that the improvement in insulin signaling by chromium was associated with the decreased IRS-1 Ser307 phosphorylation, JNK activity, and pro-inflammatory cytokine production (6). Our previous study showed that CrPic supplementation significantly enhances insulin signaling in muscle of obese JCR:LA-cp rats when compared with control rats (7). Chromium dinicocysteinate (CDNC), a complex of chromium and l-cysteine, has been noted to beneficial in lowering insulin resistance by reducing blood levels of TNF-α, insulin, and oxidative stress in type 2 diabetic subjects in a randomized, double-blind, placebo-controlled study (8). Clinical studies showed that the beneficial effects of CrPic supplementation included reduced blood glucose, insulin, cholesterol, and triglyceride levels and reduced requirements for hypoglycemic medication (9). However, these chromium supplementations (CDNC and CrPic) are only oral ones.

JDS–chromium–insulin (CRI)-003 is a novel form of insulin that has been directly conjugated with chromium (Cr) instead of zinc. CRI contains approximately 0.82% chromium by weight, which corresponds to approximately six chromium atoms per insulin hexamer unit. JDS-CRI-003 is a highly purified form of the chromium–insulin complex. Earlier studies have evaluated the activity of a crude mixture of the chromium–insulin complex, but this is the first study to evaluate the effects of the purified version of CRI.

Insulin clearance plays a major role in glucose homeostasis and insulin sensitivity in physiological and/or pathological conditions, such as obesity-induced type 2 diabetes as well as diet-induced obesity (10). Insulin clearance mostly depends on degradation by liver via the insulin-degrading enzyme (IDE) (11). IDE is a highly conserved zinc metallopeptidase that is ubiquitously distributed in human tissues and particularly abundant in the brain, liver, and muscles (12). IDE activity has been historically associated with insulin and β-amyloid catabolism. However, over the last decade, several experimental findings have established that IDE is also involved in a wide variety of physiopathological processes, including ubiquitin clearance and varicella zoster virus infection (13). IDE is believed to act as a junction point of type 2 diabetes and Alzheimer’s disease (AD) (14). The preferential affinity of this enzyme for insulin results in insulin-mediated inhibition of the degradation of other peptides such as beta-amyloid. IDE localizes primarily to the cytoplasm but in some cell types localizes to the extracellular space, cell membrane, peroxisome, and mitochondrion (15, 16). Recent study demonstrated that insulin significantly increased the mRNA levels of insulin-like growth factor 1 and insulin-like growth factor 1 receptor. These impacts could be enhanced by the addition of chromium, especially chromium chelated with small peptides (CrSP). The mRNA levels of ubiquitin were significantly reduced when cells were cultured with chromium or/and insulin (17).

Our hypothesis is that CRI may be better than regular insulin (Reg-In) in reducing plasma glucose and insulin clearance by inhibiting IDE protein expression in the liver. On the other hand, CRI may enhance insulin signaling when compared with Reg-In in diabetic animal model by altering ubiquitin-proteasome activity, it has been reported that prolong insulin treatment in adipocytes induces a decrease in IRS-1 due to an enhancement of IRS-1 degradation mediated by the proteasome pathway (18). To test this hypothesis, we measured hepatic IDE, 19S, 20S proteasome and insulin signaling proteins in KKAy diabetic mice treated with CRI, Reg-In, and vehicle saline (Sham). *In vitro* experiments also were conducted in cultured HepG2 cells to confirm the findings in animals.

## Materials and Methods

### Insulin formula

Insulin stock solutions were made at insulin concentrations of 200 U/ml using the powders of Reg-In (human insulin, Sigma lot no. 12G374G) and CRI (chromium-bound human insulin, lot no. 112017) provided by JDS Therapeutics, LLC. (Purchase, NY, USA). CRI contains 0.82% chromium (per weight of insulin). The identical concentrations of these two insulin solutions were confirmed by measuring insulin after 1:1000 dilutions with PBS using a human insulin enzyme-linked immunosorbent assay (ELISA) kit (Millipore, Billerica, MA, USA). The results showed that insulin concentrations were 194 ± 6.7 μU/ml in Reg-In and 195.3 ± 3.8 μU/ml in CRI solutions, respectively [mean ± standard error of the mean (SEM), *n* = 4, *P* = NS, Figure S1 in Supplementary Material].

### Animal study

All animal experiments were performed according to a protocol approved by the Institutional Animal Care and Use Committee of Pennington Biomedical Research Center. To determine the effects of these two types of insulin on lowering blood glucose and insulin levels in a time course after injection of insulin in KKAy mice. Twenty-four male 5-week-old KKAy mice were obtained from the Jackson Laboratory (Bar Harbor, ME, USA). After arrival, the animals were housed in two mice/cage with *ad libitum* access to rodent chow and water for a 2-week acclimation period under specific pathogen-free conditions and 12-h light–dark cycle. Then, these mice were be randomly divided into three groups (*n* = 8/group); Sham, Reg-In treated, and CRI groups, respectively. All treated groups were injected insulin at dose of 2 U/kg body weight daily, and Sham group was injected the same volume of saline for 8 weeks as insulin groups. Food intake and body weight were recorded every week.

### Blood chemistry, lipids, and hormone analysis

Pharmaceutical kinetics dynamics study of CRI was performed at week 7 by intramuscular insulin injection in KKAy mice after 2 h fasting. Blood samples were collected at 0, 0.5, 1, 2, 4, and 6 h from tail bleeding (10 μl at each time point) into microcentrifuge tubes containing 10 μl of EDTA solution, before and after injection of insulin (Sham animals were injected saline, and the other two groups were injected Reg-In or CRI at dose of 2 U/kg body weight, respectively). Plasma insulin levels were determined by human ELISA kits from Millipore (Billerica, MA, USA) and plasma glucose concentrations were measured by a colorimetric hexokinase glucose assay (Sigma Diagnostics, St Louis, MO, USA). Plasma cholesterol and triglyceride concentrations were measured by a triglyceride assay kit (Eagle Diagnostics, DeSoto, TX, USA) and a cholesterol quantitation kit (BioVision, Milpitas, CA, USA), respectively.

### Western blotting analysis

Liver lysates were prepared by homogenization in buffer A (25 mM HEPES, pH 7.4, 1% nonidet P-40 (NP-40), 137 mM NaCl, 1 mM PMSF, 10 μg/ml aprotinin, 1 μg/ml pepstatin, 5 μg/ml leupeptin) using a PRO 200 homogenizer (PRO scientific, Oxford, CT, USA). The samples were centrifuged at 14,000 × *g* for 20 min at 4°C and protein concentrations of the supernatants were determined by Bio-Rad protein assay kit (Bio-Rad laboratories, Inc., Hercules, CA, USA). Supernatants (50 μg) were resolved by 8 or 12% SDS-PAGE and subjected to immunoblotting. The protein abundance was detected with antibodies against IRS-1 p^(Tyr612)^, IRS-1, IRS-2, IR β, PI 3K, Akt2 p^(Ser474)^, 20S proteasome catalytic core (CP) β2i, Akt2 (Millipore, Temecula, CA, USA), IDE (Covance, Princeton, NJ, USA), 19S proteasome base anti-S5A/Rpn10 (Calbiochem, Gibbstown, NJ, USA), and β-actin (Thermo Scientific, Pittsburgh, PA, USA) using a Western Lightning Chemiluminescence Reagent Plus kit (PerkinElmer Life Science, Boston, MA, USA), and quantified via densitometer. All the proteins were normalized to β-actin.

### Statistical analysis

Data were expressed as mean ± SEM. Comparisons of groups were implemented by unpaired *t*-test (two-sided) or ANOVA. A *P*-value <0.05 was considered statistically significant.

## Results

**Figure 1:**
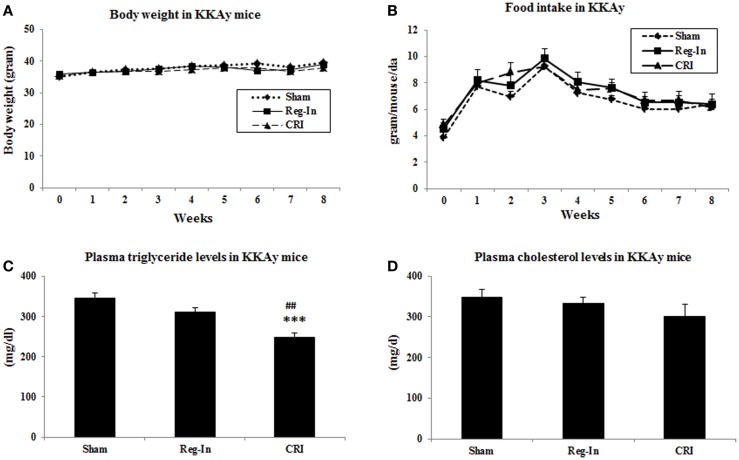
**Body weight, food intake and plasma lipids concentrations in Sham, Reg-In, and CRI treated mice**. Body weight and food intake of mice were recorded weekly. Fasting plasma lipid concentrations were measured at week 7. **(A)** Showed body weights in the mice; **(B)** is food intake in these mice; **(C)** showed plasma triglyceride concentrations; **(D)** is plasma cholesterol levels in KKAy mice. Mean ± SEM (*n* = 8/group). ***P < 0.001, CRI vs. Sham, ^##^*P* < 0.01, CRI vs. Reg-In.

Body weights, fasting plasma glucose, and insulin levels were identical in all animal groups at the beginning of study. After 8-week intervention, the body weights in Reg-In and CRI groups were slightly lower than Sham group, but there were no significant differences between these groups. Food intake was slightly lower in Sham animals than in Reg-In and CRI groups, and there were no significant differences in these groups as well (Figures 1A,B). Fasting plasma triglyceride concentrations were slightly lower in the Reg-In group than Sham group (*P* = NS), but were significantly lower in CRI animals than in Sham and Reg-In animals (*P* < 0.001 and *P* < 0.01, respectively, Figure 1C). CRI and Reg-In treatment slightly reduced plasma cholesterol levels when compared with Sham mice (*P* = NS, Figure 1D).

**Figure 2:**
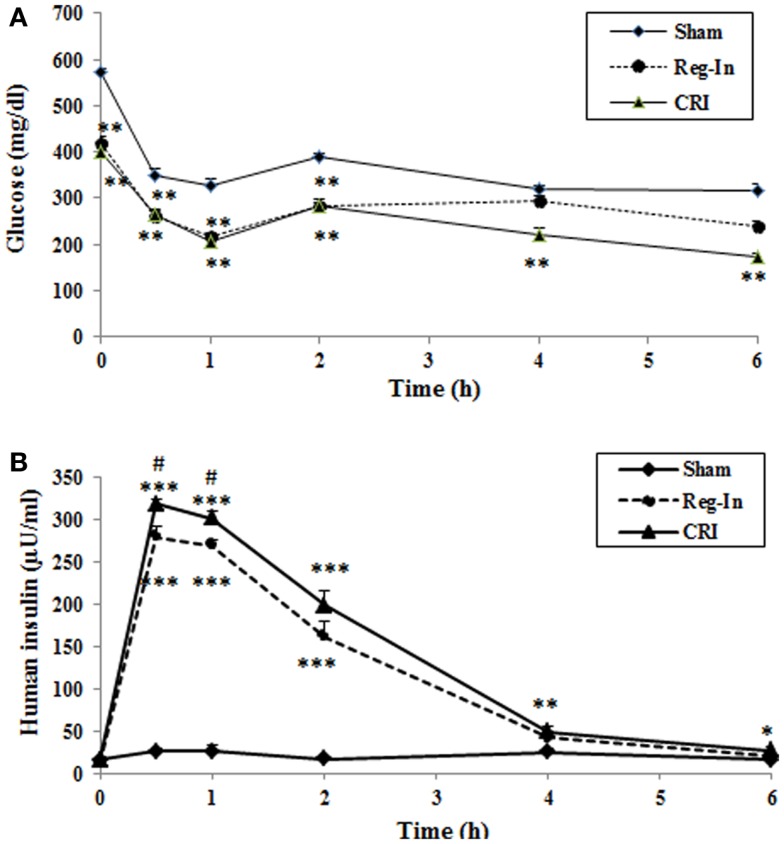
**Effects of Reg-In and CRI on glucose metabolism as well as their pharmaceutical kinetics dynamics study in KKAy mice**. At week 7, KKAy mice were sub-muscularly injected insulin at dose of 2 U/kg body weight. Blood samples were collected at various time points as indicated in the legends. Data were presented as mean ± SEM (*n* = 8/group). *P < 0.05 and **P < 0.01 and ***Sham vs. Regular-In or CRI. ^#^*P* < 0.05, ^##^*P* < 0.01 and ^###^*P* < 0.001, Reg-In vs. CRI vs. Reg-In group.

The data of pharmaceutical kinetic dynamics study showed that plasma glucose levels at baseline (0 time) were significantly lower in the Reg-In and the CRI groups than in the Sham group (*P* < 0.01). After insulin injection, the maximal effects of lowering glucose for both insulin solutions occur at 60 min. CRI significantly reduced glucose concentrations compared with Sham animals and the effect lowering glucose lasted for 6 h, but Reg-In significantly reduced glucose lasted only for 2 h (Figure 2A). Insulin pharmaceutical kinetic dynamical results showed that the insulin concentrations reach to the peak at 15 min of post-injection for Reg-In and CRI, but insulin levels at 15 and 30 min of post injection were significantly higher in CRI group than in Reg-In groups (*P* < 0.05). The insulin levels at 4 and 6 h after insulin injection were still significantly higher in CRI but not in Reg-In than in Sham animals (*P* < 0.01 and *P* < 0.05, Figure 2B).

**Figure 3:**
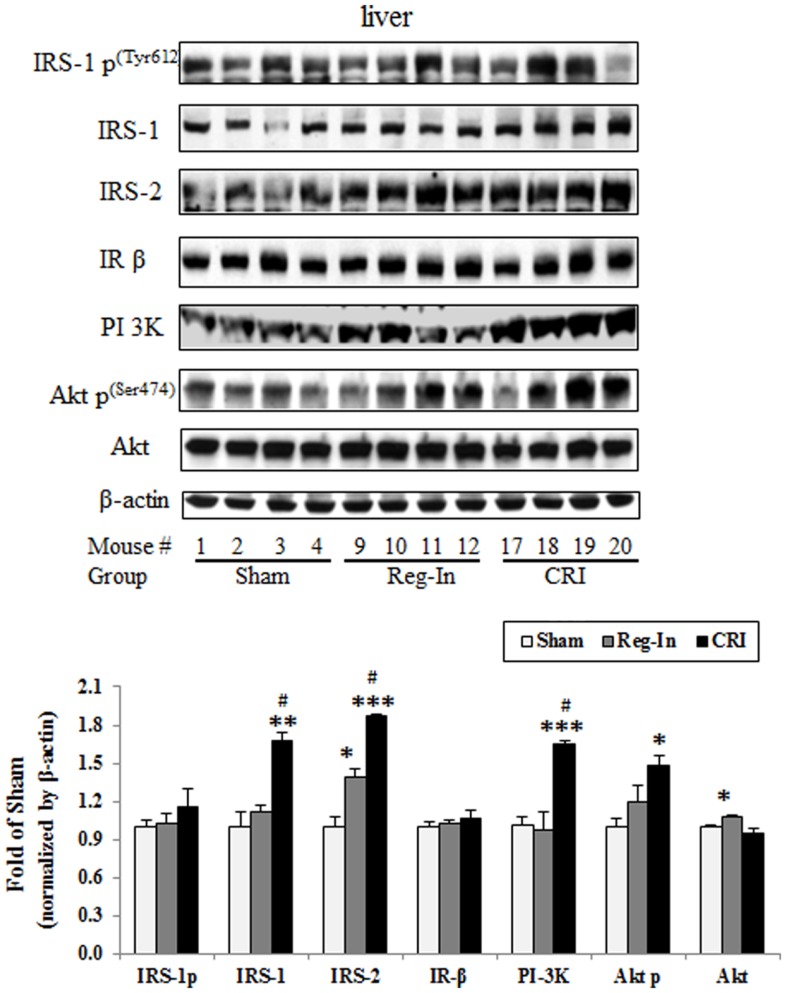
**Hepatic insulin signaling protein abundance in KKAy mice treated with or without Reg-In and CRI**. Liver lysates were subjected SDS-PAGE and transferred to nitrocellulose membranes, interesting proteins of insulin signaling were detected with specific antibodies against IRS-1, IRS-2, and so on showed in the legends. Results were normalized by β-actin. Data were presented as mean ± SEM (*n* = 8/group). *P < 0.05, **P < 0.01, and ***P < 0.001, CRI or Reg-In group vs. Sham group, respectively. ^#^*P* < 0.05, CRI vs. Reg-In.

Hepatic insulin signaling protein abundance was measured by western blotting, the data showed that CRI significantly increased hepatic IRS-1, IRS-2, PI 3K, and phosphorylation of Akt2 content in comparison to the Sham group (*P* < 0.01, *P* < 0.001, and *P* < 0.05; respectively), slightly increased IRS-1 phosphorylation without affecting IRβ and Akt2 content, while Reg-In only significantly increased Akt2, slightly increased IRS-1 and Akt2 *p* content in relative to the Sham mice, but did not alter IRS-1 *p*, IR β, and PI 3K content (Figure 3).

**Figure 4:**
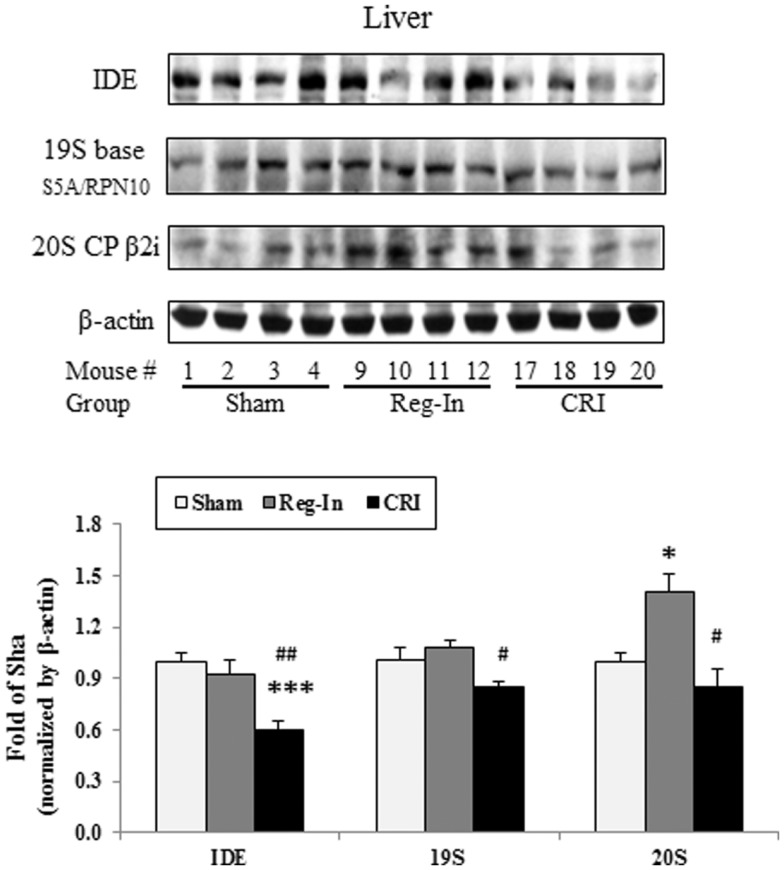
**Insulin-degrading enzyme, 19S and 20S proteasome protein abundance was analyzed in the liver of KKAy mice**. Detail was described in the Section “Materials and Methods.” Results were normalized by β-actin. Data were presented as mean ± SEM (*n* = 8/group). *P < 0.05 and ***P < 0.001, CRI or Reg-In group vs. Sham group, respectively. ^#^*P* < 0.05 and ^##^*P* < 0.01, CRI vs. Reg-In, respectively.

Chromium–insulin treatment reduced IDE and proteasome protein abundance in the liver of mice. Interestingly, CRI significantly reduced hepatic IDE abundance when compared with the Sham and Reg-In groups (*P* < 0.001 and *P* < 0.01, respectively). CRI slightly reduced 19S and 20S abundance in relative to Sham group. However, the 19S and 20S proteins in the liver were significantly lower in the CRI than in the Reg-In animals. Reg-In slightly reduced IDE content and increased 19S proteasome, but significantly increased 20S proteasome protein abundance when compared with Sham group (*P* = NS and *P* < 0.05, Figure 4). The effects of CRI on reducing IDE, 19S, and 20S proteasome protein abundance were further confirmed in HepG2 cells when compared with Reg-In (Figures S1A,B in Supplementary Material).

## Discussion

Our previous study indicates that CrPic supplementation significantly improves glucose metabolism and increases insulin sensitivity by enhancing insulin signaling in obese insulin-resistant JCR:LA-cp rats (7). However, CrPic is an oral supplementation. Using a novel form of insulin conjugated with chromium – CRI and Reg-In treated KKAy mice, we observed that both insulin treatments significantly reduced glucose concentrations and increased insulin levels compared with the Sham group, and there was no difference in glucose levels in 2 h between two insulin groups. However, CRI significantly reduced glucose concentrations at 4 and 6 h compared with Sham mice whereas Reg-In did not, suggesting that the effects of CRI on reducing glucose last longer than Reg-In. Plasma insulin concentrations at 30 and 60 min were significantly higher in the CRI group than in the Reg-In group (*P* < 0.05) as well. Compared with the Sham group, CRI treatment significantly increased hepatic insulin signaling such as IRS-1, IRS-2, PI 3K, and phosphorylation of Akt, significantly reduced IDE protein abundance (*P* < 0.01, *P* < 0.05, and *P* < 0.001, respectively), but Reg-In only significantly increased Akt1 (*P* < 0.05). The effects of CRI significantly reduced IDE content were confirmed in HepG2 cells. Similar to earlier finding that a significant reduction in serum triglyceride levels in a group of NIDDM patients treated with chromium (19), we demonstrated that CRI treatment significantly decreased plasma triglyceride concentrations compared with Reg-In and Sham groups without significantly altering plasma cholesterol levels. Moreover, Reg-In significantly increased hepatic 20S abundance mice and cultured cells relative to Sham mice and control cells, CRI greatly reduced 20S and 19S content in the liver of mice and cells in comparison with Reg-In group. Interestingly, both Reg-In and CRI treatments did not alter body weight and food intake when compared with Sham group.

One of the major findings in current study is that we are for the first time to demonstrate the effects of CRI on reduction of hepatic IDE protein abundance in a diabetic animal model. Accumulated evidence indicated that IDE has also been linked to the etiology of some diseases, such as AD and type 2 diabetes mellitus (DM2) (3), associating the protein to a wide range of cellular processes, such as, varicella zoster virus infection, steroid receptor signaling, and IDE serves as a heat shock protein with implications in cell growth regulation and cancer progression (13, 20, 21). A consequence of insulin-dependent diabetes mellitus is the loss of lean muscle mass as a result of accelerated proteolysis by the proteasome. Insulin inhibition of proteasomal activity requires interaction with IDE. Thus, IDE may function as an intracellular mediator for insulin effects on protein degradation (22). Another study suggested that a mechanism of proteasome inhibition may be the generation of inhibitory fragments of insulin, or by displacement of IDE from the proteasome (23). It is well-documented that biometals, such as copper, aluminum, and zinc, have an important role in pathological conditions of AD and diabetes mellitus. The metabolic disorders connected with these biometals lead to some metallostasis alterations in the human body and many studies point at a high level of interdependence between diabetes and several cations. Therefore, IDE activity toward Aβ peptides can be modulated by metal ions (24). The effects of different metal ions on the IDE proteolytic activity toward insulin as well as a designed peptide comprising a portion of the insulin B chain (B20–30), which has a very low affinity for metal ions. The results obtained by different experimental techniques clearly show that IDE is irreversibly inhibited by copper (I) but is still able to process its substrates when it is bound to copper (II) (25). Indeed, our study revealed that the benefits of CRI on improving glucose metabolism may contribute to reduce insulin clearance mediated by significantly decreasing hepatic IDE content *in vivo* and *in vitro* experiments (Figures 3 and 4), suggesting CRI may directly inhibit IDE as well.

**Figure 5:**
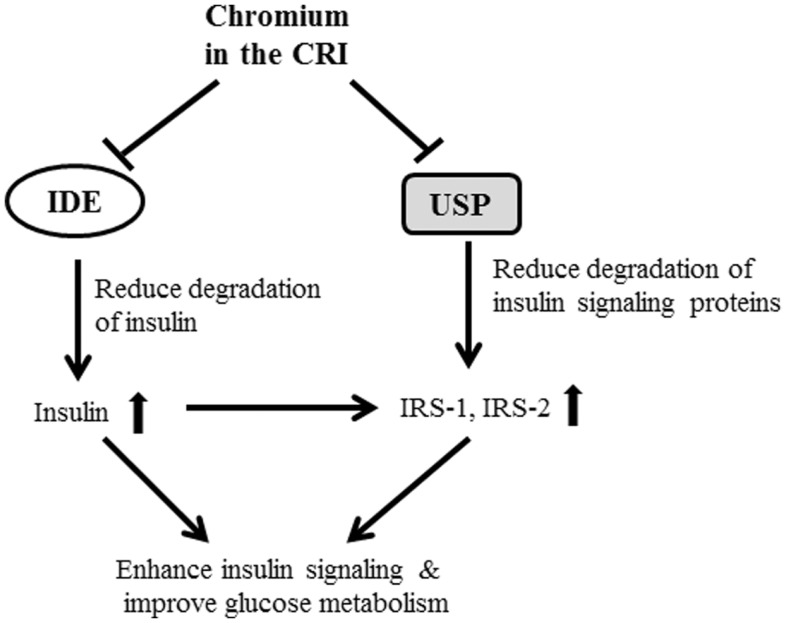
**The possible mechanisms of CRI enhancing insulin action and insulin signaling were shown in figure**. The CRI treatment may directly suppress IDE and ubiquitin-proteasome system (UPS) in the liver, and result in reduction of insulin clearance and degradation of proteins (such as IRS-1 and IRS-2) in the downstream of insulin signaling pathway. The consequent of CRI treatment enhances insulin signaling, increases insulin sensitivity, and improves glucose metabolism in insulin-resistant and diabetic conditions.

Another finding in this study is that CRI enhanced insulin signaling by suppressing 20S and 19S protein expression. It is noteworthy to mention that the 26S proteolytic complex (26S proteasome) is a macromolecular assembly thought to be involved in ATP- and ubiquitin-dependent protein degradation in the cytoplasm of higher eukaryotic cells. This complex is composed of one 20S cylinder particle, e.g., 20 core particle (CP) and two cap-shaped 19S particles, e.g., 19S regulatory particle (RP) comprising a set of polypeptides in the M(r) range of 35,000–110,000 (26–28). Dysfunctional regulation of signaling pathways downstream of the insulin receptor plays a pivotal role in the pathogenesis of insulin resistance and type 2 diabetes. Recent study shows that Cullin-RING E3 ubiquitin ligase 7 (−/−) mouse embryonic fibroblasts displayed enhanced AKT and Erk MAP kinase phosphorylation upon insulin stimulation. Depletion of CUL7 by RNA interference in C2C12 myotubes led to increased activation of insulin signaling pathways and cellular glucose uptake, as well as a reduced capacity of these cells to execute insulin-induced degradation of IRS-1 (29). In this study, we observed that CRI treatment significantly increased hepatic IRS-1, IRS-2, and PI3K protein abundance as well as significantly reduced 20S and 19S abundance in diabetic mice besides inhibition of IDE, indicating that the CRI treatment enhancing insulin signaling may be contributed to suppressing ubiquitin-proteasome activity, by which it reduces the degradation of both insulin and insulin signaling proteins (Figure 5). On the other hand, it is also interesting to investigate whether CRI is more effective than Reg-In in treatment of type 1 diabetes in the future.

## Conclusion

We observed that CRI significantly increased downstream proteins of insulin signaling pathway and decreased hepatic IDE, 19S and 20S proteasome protein abundance in KKAy mice when compared with Reg-In group. This study suggests that CRI is more effective than Reg-In in reducing glucose and attenuating insulin resistance in diabetic animal model. The possible molecular mechanism of CRI enhancing insulin action may contribute to reduce insulin clearance and protein degradation of insulin signaling by suppressing hepatic IDE and ubiquitin-proteasome pathway.

## Author Contributions

Dr. Zhong Q. Wang designed the study, wrote the manuscript, reviewed the data, and edited the manuscript. Xian H. Zhang and Yongmei M. Yu researched data. James Komorowski reviewed, edited manuscript, and provided sources of material for study; Dr. Zhong Q. Wang had full access to all the data, and takes full responsibility for the integrity of data and the accuracy of data analysis. All authors read and approved the final manuscript.

## Conflict of Interest Statement

The authors declare that the research was conducted in the absence of any commercial or financial relationships that could be construed as a potential conflict of interest.

## Supplementary Material

The Supplementary Material for this article can be found online at http://www.frontiersin.org/Journal/10.3389/fendo.2014.00099/abstract

## Abbreviations

IDE, insulin-degrading enzyme; IRS-1, insulin receptor substrate 1; UPS, ubiquitin-proteasome system.
