# Supplementary material for: Chromium–Insulin Reduces Insulin Clearance and Enhances Insulin Signaling by Suppressing Hepatic Insulin-Degrading Enzyme and Proteasome Protein Expression in KKAy Mice
Source: Front Endocrinol (Lausanne). 2014 Jul 7;5:99. doi: 10.3389/fendo.2014.00099 (PMC4083453; doi:10.3389/fendo.2014.00099)
Supplement: Supplementary file 1 [file Presentation1.PDF]

Supplementary data 1

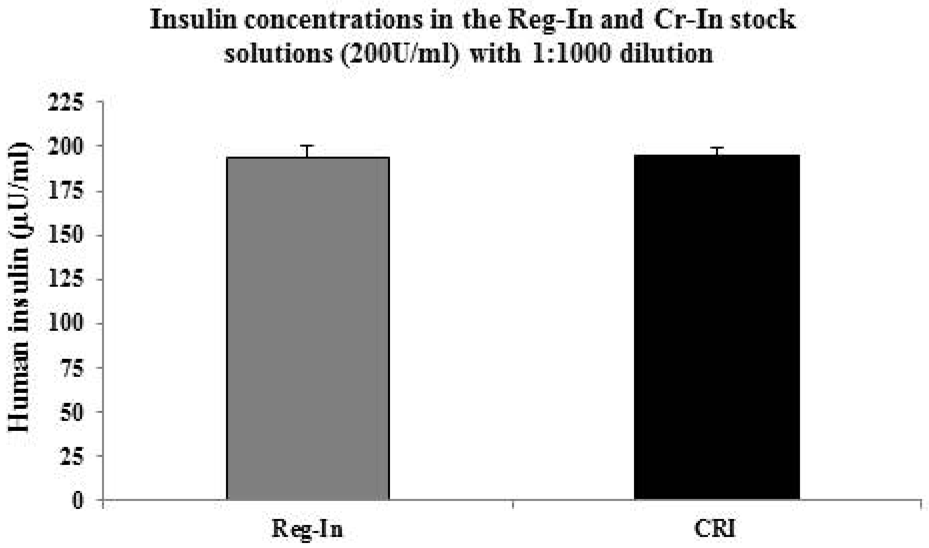

Fig 1. Stock solutions (200U/ml) of Reg-In and CRI were diluted 1:1000x with assay buffer. The data showed that there is no significant difference in insulin concentrations between two stock solutions. Mean ± SEM, for 4 measurements/sample, p=NS, Reg-In vs. CRI
