# Supplementary material for: Chromium–Insulin Reduces Insulin Clearance and Enhances Insulin Signaling by Suppressing Hepatic Insulin-Degrading Enzyme and Proteasome Protein Expression in KKAy Mice
Source: Front Endocrinol (Lausanne). 2014 Jul 7;5:99. doi: 10.3389/fendo.2014.00099 (PMC4083453; doi:10.3389/fendo.2014.00099)
Supplement: Supplementary file 2 [file Presentation2.PDF]

Supplementary data 2

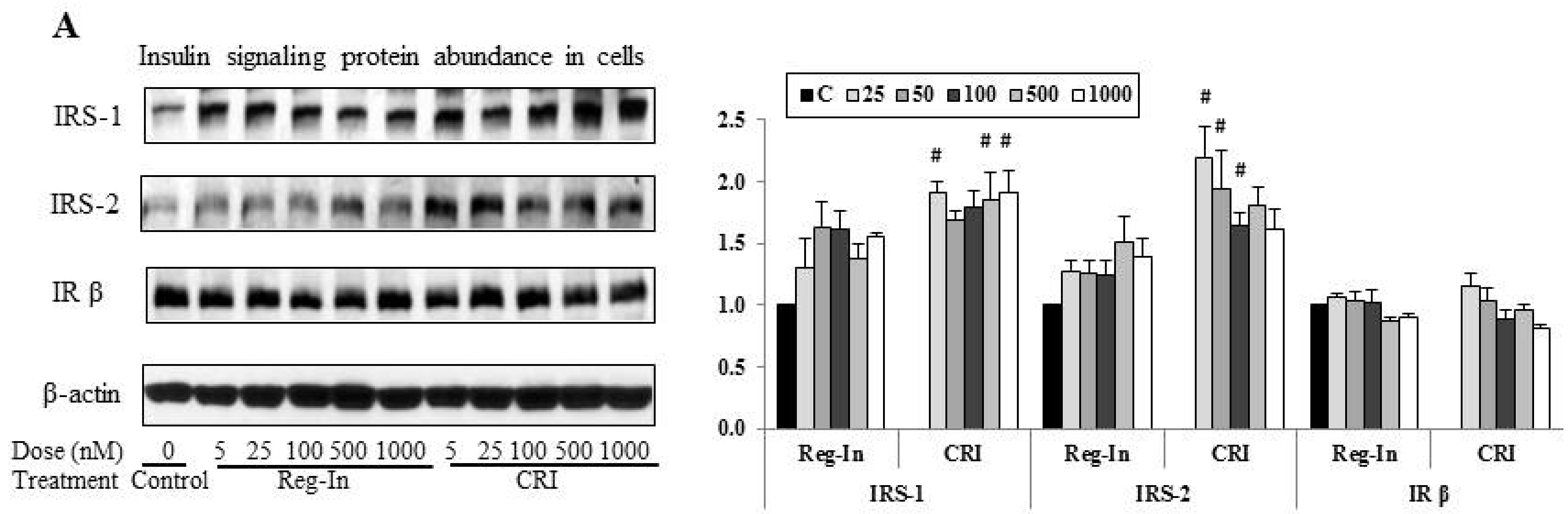

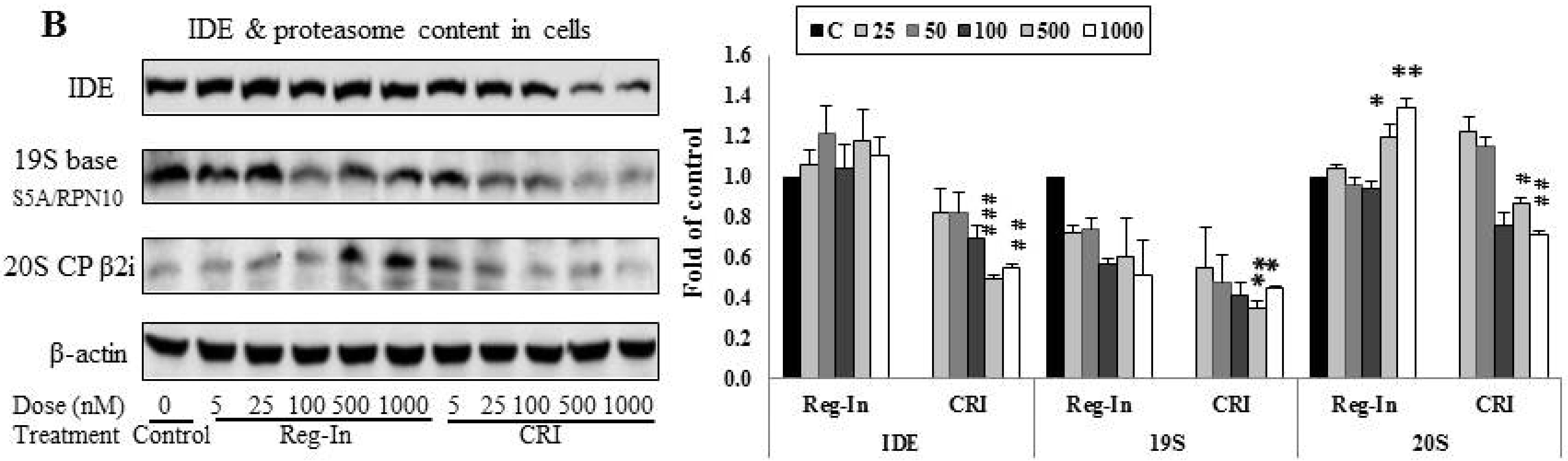

Fig 2. HepG2 cells were grown in DMEM with 10% FBS to 90% confluence, cells were maintained in DMED containing 0.2% BSA and treated with or without various concentrations of Reg-In or CRI as indicated in the legends for 24 h. Cell lysates were subjected to SDS-PAGE, insulin signaling and proteasome proteins were determined by western blotting. Data were presented as Mean ± SEM from three separated experiments, * $P<0.05$, ** $P<0.01$, CRI or Reg-In vs. control , # $P<0.05$, Reg-In vs. CRI
